# The Sublingual Microcirculation Throughout Neonatal and Pediatric Extracorporeal Membrane Oxygenation Treatment: Is It Altered by Systemic Extracorporeal Support?

**DOI:** 10.3389/fped.2019.00272

**Published:** 2019-07-10

**Authors:** Özge Erdem, Jan Willem Kuiper, Joost van Rosmalen, Robert Jan Houmes, Enno D. Wildschut, Can Ince, Dick Tibboel

**Affiliations:** ^1^Intensive Care and Department of Pediatric Surgery, Erasmus University Medical Center—Sophia Children's Hospital, Rotterdam, Netherlands; ^2^Department of Biostatistics, Erasmus University Medical Center, Rotterdam, Netherlands; ^3^Department of Intensive Care, Erasmus University Medical Center, Rotterdam, Netherlands; ^4^Department of Translational Physiology, Amsterdam University Medical Center, Amsterdam, Netherlands

**Keywords:** microcirculation, hemodynamic monitoring, extracorporeal membrane oxygenation, critical care, pediatrics, neonates

## Abstract

**Background:** Extracorporeal membrane oxygenation (ECMO) treatment alleviates systemic cardiorespiratory failure. However, it is unclear whether ECMO also improves microcirculatory function, as the microcirculation can be disturbed despite normal systemic hemodynamics. We therefore aimed to study the sublingual microcirculation (SMC) throughout neonatal and pediatric ECMO treatment. We hypothesized that the SMC improves after starting ECMO, that the SMC differs between venovenous (VV) and venoarterial (VA) ECMO, and that insufficient recovery of microcirculatory disturbances during ECMO predicts mortality.

**Methods:** This single-center prospective longitudinal observational study included 34 consecutive children (April 2016—September 2018). The SMC was assessed daily with a handheld vital microscope (integrated with incident dark field illumination) before, during, and after ECMO. Validated parameters of vessel density, perfusion, and flow quality were assessed for all vessels (diameter <100 μm) and small vessels (<20 μm). Linear mixed models and logistic regression models were built to assess changes over time and identify significant covariates. Using ROC curves, the predictive values of microcirculatory parameters were assessed for mortality on ECMO and overall mortality.

**Results:** The study population comprised 34 patients (median age 0.27 years, 16 neonates, 16 females). Twelve patients were treated with VV and 22 with VA ECMO. Twelve patients died during ECMO (stopped due to futility) and 3 died after ECMO but before discharge. Microcirculatory parameters did not change significantly before, during or after ECMO. Except between microcirculatory flow index (MFI) and mean arterial pressure (MAP), no significant associations were found between microcirculatory parameters and global systemic hemodynamics. The probability of an undisturbed MFI (>2.6) increased with higher MAP (OR: 1.050, 95%CI: 1.008–1.094). Microcirculatory parameters did not significantly differ between VV and VA ECMO or between survivors and non-survivors. None of the microcirculatory parameters could predict mortality on ECMO or overall mortality.

**Conclusion:** In this heterogeneous study population, we were not able to demonstrate an effect of ECMO on the sublingual microcirculation. Microcirculatory parameters did not change throughout ECMO treatment and did not differ between VV and VA ECMO or between survivors and non-survivors. Future research should focus on determining which neonatal and pediatric ECMO patients would benefit from microcirculatory monitoring and how.

## Introduction

Extracorporeal membrane oxygenation (ECMO) is an advanced form of mechanical life support for children with acute cardiorespiratory failure. While ECMO treatment supports systemic cardiorespiratory function, it is unclear whether it improves microcirculatory function and to what extent.

With the introduction of handheld vital microscopy, it has become possible to visualize the microcirculation in a non-invasive manner. This has offered insight into microcirculatory function, i.e., oxygen transport on a microcirculatory level. In several clinical settings, handheld vital microscopy has revealed the presence of microcirculatory disturbances despite normal systemic hemodynamics, thus possibly detecting otherwise unnoticed disturbed tissue oxygenation (1). These microcirculatory disturbances have been found to be independently associated with increased mortality risk (2–4).

The microcirculation of the sublingual mucosa has shown to be a good representation of other microvascular beds under a variety of pathophysiologic conditions (5–7). The sublingual microcirculation has shown to be predictive for survival in adult patients treated with venoarterial (VA) ECMO for cardiogenic shock and has even been proposed as a novel marker for successful weaning from VA ECMO after cardiogenic shock in adults (8, 9). Top et al. investigated the buccal microcirculation in neonates with severe respiratory failure treated with VA ECMO and showed that the buccal microcirculation was preserved after start of ECMO treatment (10). However, it remains unclear how the buccal microcirculation relates to other microvascular beds including the sublingual microcirculation. Whether ECMO treatment affects the sublingual microcirculation has not yet been investigated in the neonatal and pediatric population.

The aim of our study is to assess the effect of ECMO treatment on the sublingual microcirculation in a neonatal and pediatric population. We hypothesize that (1) the sublingual microcirculation improves after start of ECMO treatment, (2) that microcirculatory parameters differ between venovenous (VV) and VA ECMO, and (3) that insufficient recovery of microcirculatory disturbances during ECMO treatment predicts mortality.

## Methods

### Study Design, Population, and Setting

We performed a prospective longitudinal observational study at the ICU of a tertiary university children's hospital, which serves as the national ECMO center, between April 2016 and September 2018. Consecutive ECMO patients were included to acquire a representative sample of the ECMO population of our institute. The study was approved by the local medical ethical review board (Medical Research Ethics Committee Erasmus Medical Center Rotterdam). Informed consent was waived as standard therapy was monitored through non-invasive techniques in accordance with hospital guidelines. Patients aged <18 years treated with ECMO irrespective of ECMO modus (VV or VA ECMO) and primary diagnosis were included in the study. Patients needed to be fully cooperative or sufficiently sedated in order to perform microcirculatory measurements. If a patient did not meet this criterion either before or immediately after start of ECMO treatment, he or she was excluded. The primary study parameters were the microcirculatory parameters, as described below.

### Data Collection

The sublingual microcirculation was assessed with the Cytocam (Braedius Medical, Huizen, the Netherlands). The Cytocam is a handheld vital microscopy incorporated with incident dark field illumination, high magnification lenses and a computer-controlled high-resolution image sensor (11). International expert consensus guidelines were followed for both image acquisition and analysis (12, 13). The handheld probe was gently positioned on the sublingual mucosa and five clips, each with a duration of 6 s, were recorded at the following time points: before start of ECMO treatment, within 24 h after start ECMO treatment, daily during ECMO treatment, and finally after ECMO treatment was terminated up to 2 h after decannulation. Image quality was assessed using the quality score developed by Massey et al. (14). If quality criteria were not met, images were excluded from analysis. Image analysis was performed offline using semi-automated software AVA 3.2 (MicroVision Medical, Amsterdam, the Netherlands) by an experienced researcher (ÖE), who was blinded to the origin of the clips. Image analysis yielded the following functional parameters for all microcirculatory vessels (diameter <100 μm) and for all small vessels, mainly capillaries, (diameter <20 μm): total vessel density (TVD_all_/TVD_<20μ*m*_, mm/mm^2^), proportion perfused vessels (PPV_all_/PPV_<20μ*m*_, %), perfused vessel density (PVD_all_/PVD_<20μ*m*_, mm/mm^2^), microcirculatory flow index (MFI_all_/MFI_<20μ*m*_, score between 0 and 3) and heterogeneity index of flow (HI, score between 0 and 1) (12). TVD quantifies the total vessel area visible in the frame, while PPV gives the number of perfused vessels per total number of visible vessels in the frame. PVD describes the functional vessel area visible in the frame, calculated through the multiplication of TVD and PPV. MFI is a semi-qualitative score to describe the quality of flow. To obtain MFI, the screen is divided into four quadrants and each quadrant is assigned a score between 0 and 3, where 0 = no flow, 1 = intermittent flow, 2 = sluggish flow and 3 = continuous flow. The average of the scores of the four quadrants is the MFI. To account for variability between measured areas, HI can be calculated through the equation [*(highest value—lowest value)/mean value*].

Baseline and ECMO characteristics, routine clinical and hemodynamic parameters and laboratory values were collected from electronic records and the PEdiatric Logistic Organ Dysfunction 2 (PELOD-2) score, the inotrope score (IS), and the vasoactive-inotrope score (VIS) were calculated (15, 16). Also, mortality on ECMO, ICU length of stay, and survival were assessed at the end of the study period.

### ECMO Treatment

Initiating ECMO support, modus and ECMO settings were according to a well-established hospital-based ECMO protocol that did not change over time. Depending on the age of patients, cannulation in neonates was always by surgical neck dissection and adapted Seldinger technique. For pediatric patients, femoral surgical approaches with or without a neck cannula were used. The ECMO circuit comprised the Medos DP3 centrifugal pump and either the Medos HILITE LT or the iLA Activve Membrane Ventilator oxygenators. During ECMO treatment patients received continuous unfractionated heparin as anticoagulation with dose adjustments following hospital treatment guidelines.

### Statistical Analysis

Continuous data are presented as median (1st quartile−3rd quartile) and categorical data as frequency (percentage). To compare continuous variables between survivors and non-survivors, between VV and VA ECMO, and between types of primary diagnoses, the Mann-Whitney *U*-test and the Kruskal-Wallis test were used. To account for the fact that microcirculatory measurements could not always be performed on a daily basis, measurements were divided over the following time points for analysis: before start of ECMO, after start ECMO/ECMO day 1, ECMO day 2/3, ECMO day 4/5, ECMO day 6/7 and after stop ECMO treatment. If the patient was indeed measured on a daily basis from day 2 onwards, the first measurement in the 48 h was used for analysis.

For univariable analyses of repeated measures, linear mixed models were built for microcirculatory parameters and global hemodynamic parameters with time point as the single independent variable, to assess whether these parameters differed between time points. In addition, linear mixed models were built for microcirculatory parameters with time point and another independent variable to explore which baseline and ECMO demographics and global hemodynamic parameters (parameters mentioned in Tables 1–3) affected microcirculatory parameters.

**Table 1:** General demographics study population.

	**Total**	**Survivors**	**Non-survivors**	***p*-value**
Patients	34	19 (56%)	15 (44%)	
Female	16 (47%)	8 (42%)	8 (53%)	0.515
Age (years)	0.27 (0.01–2.23)	0.02 (0.00–1.47)	0.50 (0.04–2.67)	0.089
Neonate	16 (47%)	12 (63%)	4 (27%)	0.034*
Weight (kg)	5.0 (3.3–13.3)	3.5 (3.3–9.4)	9.0 (3.5–14.0)	0.190
PELOD-2 score on day start ECMO	12 (9–17)	10 (8–13)	13 (11–17)	0.228

Continuous data are presented as median (Q1-Q3), categorical data as n (%). PELOD-2 score, PEdiatric Logistic Organ Dysfunction 2 score. *p-value <0.05 is considered statistically significant.

**Table 2:** ECMO specific demographics study population.

	**Total (*n* = 34)**	**Survivors (*n* = 19)**	**Non-survivors (*n* = 15)**	***p*-value**
**Type ECMO:**				0.350
VV	12 (35%)	8 (42%)	4 (27%)	
VA	22 (65%)	11 (58%)	11 (73%)	
**Type primary diagnosis:**				0.374
Respiratory	15 (44,1%)	10 (53%)	5 (33%)	
Cardiac	13 (38,2%)	7 (37%)	6 (40%)	
ECPR	6 (17,6%)	2 (10%)	4 (27%)	
**Primary indication for ECMO:**				
Respiratory insufficiency	3 (8.8%)	2	1	
PPHN/PHT	5 (14.7%)	3	2	
MAS/PHT	3 (8.8%)	3	0	
CDH/PHT	3 (8.8%)	2	1	
Near drowning	3 (8.8%)	1	2	
Septic shock	1 (2.9%)	0	1	
Cardiac failure	4 (11.8%)	3	1	
Cardiomyopathy	4 (11.8%)	1	3	
Myocarditis	1 (2.9%)	1	0	
Cardiogenic shock	1 (2.9%)	1	0	
ECPR	6 (17.6%)	2	4	
**Duration ECMO treatment (hrs)**	142.6 (82.4–308.5)	116.5 (81.5–231.5)	242.0 (89.0–366.5)	0.215

*Continuous data are presented as median (Q1-Q3), categorical data as n (%). CDH, congenital diaphragmatic hernia; ECMO, extracorporeal membrane oxygenation; ECPR, extracorporeal cardiopulmonary resuscitation; MAS, meconium aspiration syndrome; PHT, pulmonary hypertension; PPHN, persisting pulmonary hypertension in the newborn; VA, venoarterial; VV, venovenous. p-value <0.05 is considered significant*.

**Table 3:** Observed data per time point.

**Parameter**	**Before start ECMO (*n* = 13)**	**After start ECMO/on day 1** **(*n* = 29)**	**ECMO day 2 or 3** **(*n* = 24)**	**ECMO day 4 or 5** **(*n* = 9)**	**ECMO day 6 or 7** **(*n* = 7)**	**After stop ECMO** **(*n* = 11)**	***p*-value**
TVD_all_ (mm/mm^2^)	24.59 (20.71–26.41)	25.48 (23.15 - 28.35)	24.47 (23.13- 26.75)	28.15 (26.80 - 30.09)	27.95 (23.36 - 31.80)	25.35 (23.61 - 26.75)	0.374
PPV_all_ (%)	98.78 (96.49 - 99.16)	99.12 (97.43–99.78)	98.82 (97.27–100)	99.27 (98.01–99.75)	99.74 (98.88–99.90)	99.23 (97.76–100)	0.569
PVD_all_ (mm/mm^2^)	24.03 (20.14–26.14)	25.38 (23.10–27.99)	24.38 (22.92–25.82)	28.07 (25.90–29.92)	27.43 (23.31–31.71)	25.26 (23.11–26.30)	0.371
MFI_all_	2.78 (2.48–2.87)	2.87 (2.76- 3)	2.83 (2.73–3)	2.85 (2.64–3)	2.92 (2.64–3)	2.89 (2.73–2.98)	0.880
HI_MFI_	0.09 (0.09–0.17)	0.07 (0–0.13)	0.09 (0–0.11)	0.13 (0–0.13)	0.04 (0–0.10)	0.05 (0.02–0.15)	0.728
TVD_<20μ*m*_ (mm/mm^2^)	21.70 (18.80–23.59)	22.75 (20.68–24.68)	21.47 (20.41–23.90)	26.41 (24.30–26.90)	25.83 (20.63–28.99)	21.91 (20.51–24.23)	0.378
PPV_<20μ*m*_ (%)	98.62 (96.17–99.40)	99.08 (97.35–99.74)	98.70 (96.93–100)	99.14 (97.87–99.70)	99.71 (98.73–99.89)	99.13 (97.35–100)	0.579
PVD_<20μ*m*_ (mm/mm^2^)	21.22 (18.45–23.35)	22.18 (20.40–24.35)	21.16 (19.67–23.40)	24.48 (24.23–26.73)	25.31 (20.58–28.91)	21.65 (19.52–24.04)	0.388
MFI_<20μ*m*_	2.38 (2.06–2.71)	2.75 (2.50–3)	2.67 (2.50–3)	2.67 (2.33–3)	2.75 (2.33–3)	2.75 (2.44–3)	0.381
MAP (mmHg)	55 (42–70)	57 (48–66)	58 (51–71)	48 (46–59)	55 (43–58)	47 (38–52)	0.053
HR (bpm)	157 (136–166)	147 (121–156)	134 (106–150)	119 (114–123)	131 (127–147)	145 (122–154)	0.003*
CVP (mmHg)	9 (6–10)	11 (5–17)	10 (6–18)	(–)	(–)	(–)	0.587
Fluid balance (ml/kg/d)	42 (8–75)	40 (26–69)	−11 (-40–65)	12 (−26–116)	−18 (−21–61)	9 (−4–44)	0.193
pH	7.26 (7.18–7.34)	7.34 (7.23–7.38)	7.36 (7.33–7.42)	7.33 (7.32–7.42)	7.41 (7.29–7.47)	7.29 (7.22–7.44)	0.205
PaO_2_ (kPa)	11.3 (7.0–18.5)	10.8 (8.0–17.2)	8.7 (6.8–11.3)	10.3 (8.9–11.6)	11.1 (6.9–18.3)	16.7 (11.3–20.9)	0.167
PaCO_2_ (kPa)	6.4 (5.2–9.1)	5.7 (5.0–6.3)	5.8 (5.5–6.1)	5.6 (5.3–6.6)	5.5 (5.3–7.1)	6.1 (5.2–8.0)	0.017*
SaO_2_ (%)	90 (83–99)	96 (93–100)	95 (89–98)	97 (93–98)	98 (96–100)	99 (98–100)	0.008*
Hemoglobin (mmol/L)	7.6 (5.9–8.8)	6.7 (6.0–7.7)	7.5 (6.9–7.9)	6.7 (6.5–7.3)	6.5 (6.3–6.9)	6.5 (6.4–6.8)	0.085
Hematocrit (L/L)	0.35 (0.25–0.39)	0.33 (0.29–0.36)	0.34 (0.32–0.36)	0.32 (0.30–0.32)	0.33 (0.30–0.34)	0.32 (0.29–0.35)	0.838
Lactate (mmol/L)	4.1 (1.4–6.0)	3.6 (1.9–8.5)	1.4 (1.2–3.2)	1.3 (1.0–4.9)	1.5 (0.8–3.5)	0.8 (0.6–1.3)	<0.001*
IS	12 (0–48)	10 (0–38)	10 (0–24)	0 (0–5)	0 (0–0)	0 (0–4)	0.017*
VIS	72 (0–98)	27 (5- 80)	10 (0–36)	0 (0–5)	2 (0–49)	5 (2–14)	0.002*
On ECMO	0 (0%)	34 (100%)	31 (91%)	22 (64%)	17 (50%)	0 (0%)	NA
Mortality	0 (0%)	0 (0%)	2 (6%)	4 (12%)	5 (15%)	12 (35%)	NA

Table shows the observed raw data per time point. The last column shows the p-values based on the univariable linear mixed models. Continuous data are presented as median (Q1-Q3), categorical data as n (%). CVP, central venous pressure; ECMO, extracorporeal membrane oxygenation; HI, heterogeneity index of flow; HR, heart rate; IS, inotrope score; MAP, mean arterial pressure; MFI, microcirculatory flow index; NA, not applicable; PaCO_2_, partial arterial carbon dioxide pressure; PaO_2_, partial arterial oxygen pressure; PPV, proportion perfused vessels; PVD, perfused vessel density; SaO_2_, arterial oxygen saturation; TVD, total vessel density; VIS, vasoactive inotrope score; * significant change over time points (univariable linear mixed models, p <0.05).

For multivariable analyses of repeated measures, linear mixed models (continuous outcome parameter: PVD) and logistic regression models based on generalized estimating equations with a binomial error distribution and logit link function (categorical outcome parameter: MFI >2.6) were built. MFI ≤2.6 has been proposed and shown, but not yet validated in children, as the threshold to identify a disturbed microcirculation (3, 4). Significant independent variables from the univariable analyses were tested in these multivariable analyses. The independent variables tested for PVD_all_ and PVD_<20μ*m*_: pediatric patient, sex, sildenafil treatment, plasma transfusion in the last 24 h, crystalloids transfusion in the last 24 h, partial O_2_ pressure (PaO_2_, kPa), and norepinephrine (μg/kg/min); for MFI_all_:, pediatric patient: sex, PaO_2_ (kPa), norepinephrine (μg/kg/min), duration ECMO treatment (hours), mean arterial pressure (MAP, mmHg), hemoglobin (L/L), and fraction inspired O_2_ (FiO_2_, %) of the sweep gas flow; for MFI_<20μ*m*_: pediatric patient, norepinephrine (μg/kg/min), FiO_2_ (%), MAP (mmHg), hemoglobin (L/L), and FiO_2_ of the sweep gas flow (%). The stepwise backward method with a *p*-value selection threshold of 0.2 was then applied to obtain the final multivariable model. Time point was forced into both univariable and multivariable models, irrespective of the *p*-value. A random intercept was included in the linear mixed models to account for the within-subject correlations. The linear mixed models account for missing data in the outcome and no form of data imputation was used.

Receiver-operating-characteristic (ROC) curves were built to assess the predictive value of microcirculatory parameters per time point for mortality on ECMO and overall mortality. Two-sided *p*-values lower than 0.05 were considered statistically significant, but a Bonferroni-adjusted significance level of 0.008 was used for the ROC analyses. Statistical analysis was performed in IBM SPSS statistics 24 (IBM, Armonk, NY, USA).

## Results

### Demographics

**Figure 1:**
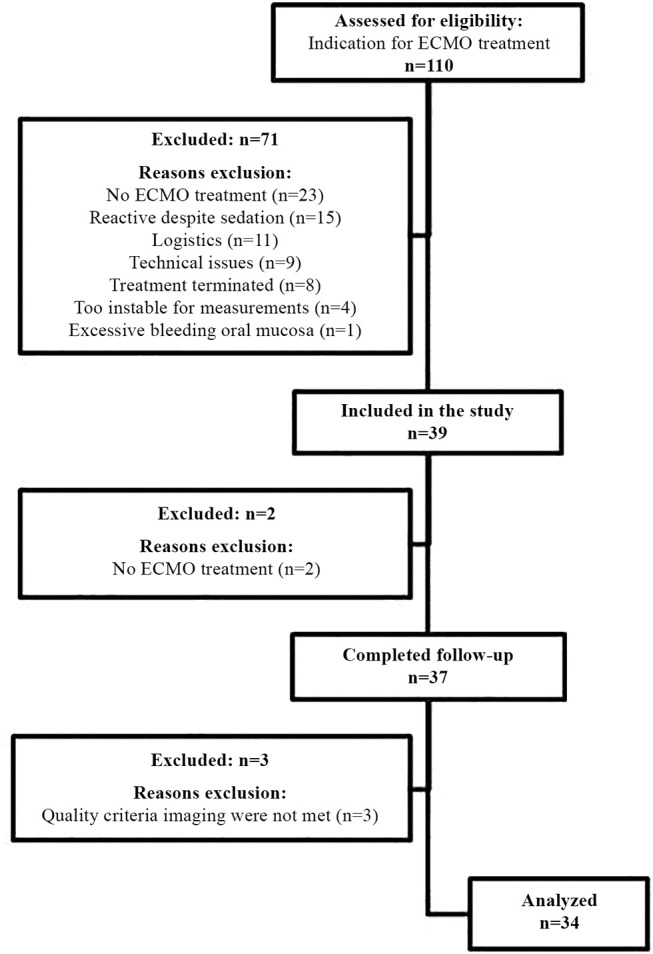
Flowchart inclusion study population.

Figure 1 shows the flow chart for inclusion. Thirty-seven consecutive patients were included in the study. Three patients were excluded from analysis as the collected images did not meet quality criteria. Table 1 describes the general demographics of the study population. The study population comprised 34 patients, with a median age of 0.27 years, 16 neonates (47%), 16 females (47%), and a median PELOD-2 score of 12 (mortality risk of >30%). Table 2 describes ECMO-specific demographics. Twelve patients (35%) received VV ECMO treatment and 22 patients (65%) VA ECMO treatment. Primary indications for ECMO treatment included 15 respiratory diagnoses (44%), 13 cardiac diagnoses (38%), and 6 extracorporeal cardiopulmonary resuscitation (ECPR) cases (18%). Four of the 13 cardiac diagnoses (12%) were cyanotic heart diseases. The median duration of ECMO treatment was 142.6 h. Type of ECMO, primary indications for ECMO treatment, and duration of ECMO treatment did not significantly differ between survivors and non-survivors.

### Univariable Analyses of Microcirculatory and Global Hemodynamic Parameters

Table 3 shows the microcirculatory and global hemodynamic parameters per time point. Microcirculatory measurements were possible in 38% of the study population before start of ECMO treatment, in 85% after start of ECMO treatment, in 77% on day 2 or 3, in 41% on day 4 or 5 and on day 6 or 7 and in 50% after successful weaning from ECMO. Microcirculatory parameters did not change after start of and during ECMO treatment, while global hemodynamic parameters did show improvement over time. Heart rate, partial CO_2_ pressure (PaCO_2_), serum lactate, IS and VIS decreased over time, while arterial O_2_ saturation increased. Microcirculatory parameters did not significantly differ between VV and VA ECMO or between types of primary diagnosis. In addition, microcirculatory parameters were not different for patients with cyanotic heart disease. The 62% of ECMO patients who could not be measured before the start of ECMO treatment did not have different microcirculatory parameters at the consecutive time points than the 38% of ECMO patients who were measured before ECMO was initiated (*p* > 0.05). No differences could be found between the two groups in PELOD-2 score, type of ECMO, primary indication for ECMO, and mortality rates (*p* > 0.05).

### Multivariable Analyses of Microcirculatory Parameters PVD and MFI

Table 4 summarizes the results of the linear mixed models for PVD. Both PVD_all_ and PVD_<20μ*m*_ were lower if the patient was a pediatric patient (> 28 days old) or female or had received plasma transfusion in the last 24 h before measurement. Both PVD_all_ and PVD_<20μ*m*_ were lower if continuous intravenous infusion of norepinephrine was administered at a higher dose or if PaO_2_ increased. PVD was higher if the patient received sildenafil or crystalloids in the last 24 h before measurement. Table 5 summarizes the results of the logistic regression models for MFI > 2.6. The probability of MFI_all_ > 2.6 was lower in pediatric patients than in neonates. The probability of MFI_all_ > 2.6 was higher when patients were on ECMO for a longer period, MAP was higher, or FiO_2_ of the sweep gas flow was set higher. The probability of MFI_all_ > 2.6 was lower when PaO_2_ was higher. The probability of MFI_<20μ*m*_ > 2.6 was higher with higher MAP or FiO_2_ but decreased with higher hemoglobin levels.

**Table 4:** Multivariable linear mixed models for PVD_all_ and PVD_<20μ*m*_.

**Repeated measures mixed model: PVD_**all**_**	**Estimate B**	**95% CI**	***p*-value**
Intercept	28.321	24.214–32.429	<0.001
Time point			0.188
Before start ECMO	1.737	−1.242–4.715	0.247
After start ECMO/on day 1	2.856	0.441–5.270	0.021
ECMO day 2 or 3	1.012	−1.550–3.574	0.431
ECMO day 4 or 5	1.455	−1.529–4.439	0.332
ECMO day 6 or 7	2.232	−1.025–5.488	0.174
After stop ECMO	0 (reference)	–	–
Pediatric patient (>28 days old) (c)	−2.294	−4.381–0.207	0.032
Male (c)	1.490	−0.486–3.466	0.134
PaO_2_ (kPa)	−0.118	−0.223–0.012	0.029
No sildenafil treatment (c)	−2.165	−4.724–0.394	0.095
No plasma transfusion in last 24 h (c)	1.339	−0.412–3.091	0.131
No crystalloids administration in last 24 h (c)	−2.400	−4.342–0.458	0.016
Dose continuous IV norepinephrine (μg/kg/min)	−3.996	−7.417–0.580	0.023
**Repeated measures mixed model: PVD**_**<20μ*m***_	**Estimate B**	**95% CI**	***p*****-value**
Intercept	26.017	22.097–29.936	<0.001
Time point			0.077
Before start ECMO	2.657	−0.210–5.524	0.069
After start ECMO/on day 1	3.627	1.300–5.955	0.003
ECMO day 2 or 3	2.043	−0.427–4.513	0.103
ECMO day 4 or 5	1.840	−1.034–4.714	0.205
ECMO day 6 or 7	2.518	−0.623–5.659	0.113
After stop ECMO	0 (reference)	–	–
Pediatric patient (>28 days old) (c)	−3.192	−5.159–1.225	0.002
Male (c)	1.260	−0.597–3.116	0.176
PaO_2_ (kPA)	−0.132	−0.233–0.030	0.012
No sildenafil treatment (c)	−2.783	−5.201–0.365	0.025
No plasma transfusion in last 24 hours (c)	1.716	0.032–3.399	0.046
No crystalloids administration in last 24 hours (c)	−2.727	−4.590–0.865	0.005
Dose continuous IV norepinephrine (μg/kg/min)	−3.667	−6.931–0.404	0.028

*C, categorical variable; CI, confidence interval; ECMO, extracorporeal membrane oxygenation; PaO_2_, partial O_2_ pressure; PVD, perfused vessel density*.

**Table 5:** Repeated measures logistic regression (GEE) models for MFI_all_ > 2.6 and MFI_<20μ*m*_ > 2.6.

**Repeated measures logistic regression models: MFI_**all**_ > 2.6**	**Odds ratio**	**95% CI**	***p*-value**
Intercept (baseline odds)	0.652	0.018–23.460	0.815
Time point			0.336
Before start ECMO	0.188	0.017–2.127	0.188
After start ECMO/on day 1	0.045	0.003–0.663	0.045
ECMO day 2/3	0.036	0.002–0.667	0.036
ECMO day 4/5	0.021	0.000–1.327	0.021
ECMO day 6/7	0.064	0.003–1.199	0.064
After stop ECMO	1 (reference)	–	–
Pediatric patient (>28 days old) (c)	0.311	0.063–1.536	0.152
Duration ECMO (hrs)	1.009	1.002–1.015	0.009
MAP (mmHg)	1.068	0.989–1.153	0.093
PaO_2_ (kPa)	0.892	0.836–0.953	0.001
FiO_2_ of sweep gas flow ECMO (%)	1.026	0.997–1.057	0.077
**Repeated measures logistic regression models: MFI**_**<20μ*m***_ **>** **2.6**	**Odds ratio**	**95% CI**	***p*****-value**
Intercept (baseline odds)	1.843	0.025–135.438	0.780
Time point			0.149
Before start ECMO	0.047	0.004–0.583	0.017
After start ECMO/on day 1	0.449	0.097–2.070	0.304
ECMO day 2/3	0.204	0.026–1.612	0.132
ECMO day 4/5	0.354	0.033–3.831	0.392
ECMO day 6/7	0.692	0.065–7.424	0.761
After stop ECMO	1 (reference)	–	–
FiO_2_ (%)	1.022	0.996–1.048	0.101
Hemoglobin (mmol/L)	0.612	0.354–1.057	0.078
MAP (mmHg)	1.050	1.008–1.094	0.019

*C, categorical variable; CI, confidence interval; FiO_2_, fraction inspired oxygen; MAP, mean arterial pressure; MFI, microcirculatory flow index; PaO_2_, partial oxygen pressure*.

### Outcome

Twelve patients (35%) died during ECMO treatment, as ECMO treatment was stopped due to futility, and 3 patients (9%) died after ECMO treatment before ICU discharge. Leading causes of death during ECMO treatment were multi-organ failure (*n* = 5), severe brain damage and multi-organ failure (*n* = 4), severe brain damage (*n* = 2), and no treatment options (*n* = 1). Leading causes of death after ECMO treatment were no therapeutic options (*n* = 1), severe brain damage (*n* = 1) and untreatable pulmonary hypertension (*n* = 1). The median ICU length of stay was 15 days (11–23). Before start of ECMO treatment, there were no significant differences between survivors and non-survivors in global systemic hemodynamics or need for vasoactive drugs. Microcirculatory parameters did not differ between survivors and non-survivors on all time points separately. None of the microcirculatory parameters measured before start of ECMO treatment, after start of ECMO treatment or on day 2 or 3 of ECMO treatment could predict mortality on ECMO or overall mortality as none of the parameters had a significant area under the curve in the ROC curve analysis, summarized in Table S1.

## Discussion

In our study with consecutively included patients, an effect of ECMO treatment on the sublingual microcirculation could not be demonstrated. Microcirculatory parameters did not change immediately after starting ECMO, nor during or after ECMO treatment. Microcirculatory parameters did not significantly differ between VV and VA ECMO. Multivariable analyses showed that PVD values were influenced by patients' age, sex, plasma transfusion, infusion of norepinephrine, PaO_2_ levels, sildenafil treatment, and resuscitation with crystalloids. The probability of having an MFI > 2.6 was influenced by patients' age, MAP, FiO_2_ settings, PaO_2_ levels, and hemoglobin levels. Additionally, microcirculatory parameters did not significantly differ between survivors and non-survivors and did not predict mortality on ECMO or overall mortality.

In contrast to our hypothesis, the microcirculation did not change after the initiation of ECMO treatment in our study population, despite the effects of ECMO on systemic hemodynamics. ECMO treatment was started in patients with cardiovascular and/or respiratory failure when conventional treatment fell short in the support of global systemic hemodynamics. After ECMO initiation global systemic hemodynamics improved and the need for vasoactive and inotropic drugs and fluid resuscitation decreased. However, microcirculatory parameters appeared largely unaffected both before and immediately after initiation of ECMO treatment. As shown, PPV and MFI values were high before initiation of ECMO treatment and remained high at consecutive time points. Statistical analysis did not reveal specified patient groups in which the microcirculation was compromised before the initiation of ECMO treatment or changed after initiation of ECMO treatment. In contrast to adult studies in which the microcirculation improved in patients with cardiogenic shock treated with VA ECMO, type of ECMO and type of primary diagnosis (circulatory vs. respiratory) did not define groups in which ECMO treatment improved the microcirculation (8, 9). Similarly, nor PELOD-2 score, IS or VIS defined a specific group with an affected microcirculation that improved during ECMO treatment. There are several possible explanations for why the microcirculation was unaffected by ECMO treatment in our study. Due to clinical instability and technical and logistic issues, the microcirculation could only be assessed in 38% of patients before the initiation of ECMO. This possible selection bias could have affected the results. However, the patients who were not measured before ECMO treatment did not differ from the patients who were measured based on PELOD-2 score, type of ECMO, the primary indication for ECMO, and overall mortality. Additionally, the microcirculation did not differ at consecutive time points between these two groups. This suggests that the possible selection bias does not explain the lack of microcirculatory compromise and subsequent absence of change in the microcirculatory parameters over time in our study. Another explanation would be that ECMO treatment may have been initiated before global systemic hemodynamics deteriorated and influenced microcirculatory parameters. Also, despite critical illness and altered global systemic hemodynamics, which both affect microcirculatory function, the microcirculation has auto-regulatory mechanisms to preserve blood flow and tissue oxygenation (17). These explanations are supported by the relatively low lactate levels at the start of ECMO treatment. Finally, our study population may have been too small or heterogeneous to show effects or observe differences over time. However, this is not the first study to find an unaltered microcirculation during ECMO treatment. Similar to our results, in neonates with severe respiratory failure treated with VA ECMO, Top et al. reported that the PVD of the buccal microcirculation was unaltered after ECMO treatment was started (10). In contrast to our findings, Top et al. also found that after successful weaning from ECMO PVD was higher than after starting ECMO treatment (18).

In contrast to our expectations, microcirculatory parameters did not significantly differ between VV and VA ECMO. VV ECMO is generally started for respiratory insufficiency. As VA ECMO is started for cardiac failure or cardiorespiratory failure, more effects on the microcirculation were expected both before and after the initiation of VA ECMO. After starting VV ECMO treatment, hypoxia was resolved quickly. Similarly, hemodynamic instability was more often than not resolved quickly after starting VA ECMO, apparent by declining need of inotropic and vasoactive drugs. Then, for both types of ECMO, global systemic hemodynamics are restored before the microcirculation could deteriorate or the microcirculation is preserved despite altered global systemic hemodynamics. Another explanation could be that patients with pulmonary hypertension [*n* = 5 (42% of VV ECMO patients)] treated with VV ECMO are more comparable to VA ECMO patients than one would expect. Patients with pulmonary hypertension can present with hemodynamic instability, not different from patients with cardiac failure. However, these patients with pulmonary hypertension are treated with VV ECMO as this is often times sufficient to resolve the pulmonary hypertension and indirectly the hemodynamic instability that goes along with it (19, 20). These VV ECMO patients and VA ECMO patients before treatment might then have similar hemodynamics and similar microcirculatory properties while being treated with different ECMO entities.

PVD is used to assess the functional vessel density of the microcirculation, a surrogate for the diffusive capacity, i.e., the distance O_2_ covers from the RBC to the tissue cell (12). Multivariable analyses showed that age group, sildenafil treatment, and resuscitation with crystalloids had the biggest impact on PVD. PVD was lower in pediatric patients than in neonatal patients and in females than in males. This aligns with findings from several observational studies performed in healthy neonates and children, wherein these studies vessel density of cutaneous and buccal microcirculation declined over the first few weeks of life and vessel density of cutaneous microcirculation was lower in females than in males (21–24). Our comparable findings support the accuracy of the technique used, because, despite these being highly complex and critically ill children, the handheld vital microscopy yielded results in which the effect of age and sex on the microcirculation could still be distinguished from the other multiple factors affecting the microcirculation. A possible explanation for age-related differences is the developmental changes of the cardiovascular system, which have not yet been thoroughly investigated for the microcirculation (25). PVD was also higher in (neonatal) patients treated with sildenafil for pulmonary hypertension. As sildenafil decreases vascular resistance through vasodilation, blood flow through capillaries increases, which may account for the increase of PVD. Similar effects were assessed with handheld vital microscopy in an animal study on sildenafil treatment and cardiorespiratory resuscitation by Wu et al. (26). Although our data also suggest that norepinephrine decreases PVD, this effect was only clinically relevant if high doses (>1.0 μg/kg/min) were administered. This might explain why Buijs et al. found that catecholaminergic drugs, including norepinephrine, improved global systemic hemodynamics but did not alter the microcirculation in neonates, as patients in their study only received a median dose of 0.11 μg/kg/min (IQR: 0.3) (27).

MFI is a qualitative measure of microvascular blood flow velocity and describes the convective component of the microcirculation (12). An MFI ≤2.6 is considered to be disturbed flow, though this has not yet been validated in children (3, 4). In our study, MAP and PaO_2_ levels had the biggest influence on MFI_all_, while hemoglobin levels had the biggest impact on MFI_<20μ*m*_. MAP was also found to influence the quality of flow and, thus, there seemed to be no loss of hemodynamic coherence throughout ECMO treatment. As systemic hemodynamics improved as MAP increased, this increased the probability of having an MFI >2.6. Interestingly, our study also found to have decreased probabilities for having an MFI >2.6 with higher PaO_2_ levels, while having higher probabilities with higher FiO_2_. One would expect a patient with the need for higher FiO_2_ to have lower saturations and lower PaO_2_ values, given that other hemodynamic parameters are unaltered. However, it is not understood why a patient who requires higher FiO_2_ and has lower PaO_2_ would be more likely to have a better blood flow quality compared to a patient who requires less FiO_2_ and has higher PaO_2_ levels. Finally, increasing levels of hemoglobin increased the probability of having an MFI >2.6. A similar finding was reported in an animal study, in which after blood transfusions hemoglobin increased concomitantly with increasing renal blood flow velocity (28). In contrast, RBC transfusions in anemic preterm infants and children improved both hemoglobin levels and PVD but did not affect MFI values (29, 30).

While previous studies performed in adults demonstrated the predictive value of microcirculatory parameters for outcome, our study was not able to reach the same conclusion. The sublingual microcirculation did not significantly differ between survivors and non-survivors. Subsequently, sublingual microcirculatory parameters before starting ECMO treatment, after starting ECMO treatment, or on day 2 or 3 could not predict mortality on ECMO or overall mortality. Although we expected that the microcirculation in non-survivors would be compromised before initiation of ECMO and would not improve, mortality may not always be reflected in microcirculatory changes caused by the underlying disease. In 24 adults with cardiogenic shock treated with VA ECMO, Kara et al. showed that PVD differed between survivors and non-survivors at all-time points (8). Moreover, in this small group of patients, PVD after start ECMO treatment could predict survival. In a similar population of 48 adults, Yeh et al. found differences in PVD and PPV between survivors and non-survivors (31). The aforementioned studies only included patients with cardiogenic shock, while our study had a heterogeneous population with fewer cases of cardiogenic shock. The MicroSOAP study, an international study with a heterogeneous adult ICU study population with over 500 patients showed that an abnormal MFI was independently associated with increased mortality risk if patients presented with tachycardia (3). Similar observations could not be made in our study. Our study may not have had sufficient power to test additional parameters for their predictive value. However, to date there have been no studies on the predictive value of microcirculatory parameters in children, it is therefore uncertain if the microcirculation acts differently in children than in adults. This could be especially true in neonates, as they undergo developmental changes that have not yet been fully investigated (21–23). Furthermore, in our study, death was not always a result of cardiorespiratory insufficiency but could also have resulted from irreversible cerebral damage [*n* = 7 (47%)] or lack of further therapeutic options [*n* = 3 (20%)], such as with refractory pulmonary hypertension or severe heart failure without the option for heart transplantation. These deceased patients did not necessarily have a disturbed microcirculation, which is supported by the finding that the medians and interquartile ranges of microcirculatory parameters of our study population were higher than those of the previously mentioned adult study populations (8, 31). It was therefore unlikely that microcirculatory alterations could predict outcome in this study population.

Whether microcirculatory parameters could also be used in children as a biomarker for successful weaning from ECMO treatment remains unclear. Microcirculatory parameters measured before or after starting ECMO treatment, or on day 2 or 3 could not predict whether a patient could eventually be successfully weaned from ECMO treatment. We were not able to use microcirculatory parameters as a dynamic parameter to assess whether patients could be successfully weaned, as Akin et al. did in adults (9). Measurements could often not be performed during the desired moment to attempt weaning, as patients were awake. Also, while some institutions routinely perform trial offs to assess whether patients can be weaned from ECMO, our institution gradually decreases ECMO flow rate over time to eventually clamp the ECMO circuit and wean patient from ECMO support.

Our study has some limitations that deserve mention. Although handheld vital microscopy is non-invasive and relatively easy to apply, practical issues account for the missing values at different time points. The feasibility of repeated microcirculatory measurements was limited, as our study population comprised mostly of young patients. It is challenging for young children, while being awake, to remain motionless with their mouth and tongue for several seconds during measurements, causing both movement and pressure artifacts in the recorded imaging. This is especially the case for neonates, as they exhibit a sucking reflex. Often times patients were comfortably sedated but awake, did not cooperate or were too unstable clinically to perform measurements with adequate image quality. This led to the exclusion of 36% of the eligible patients and to missing data in the study population. These challenges are not unique to our study. Gonzalez et al. showed that in a general pediatric ICU population, microcirculatory assessment was only possible in 17% of the patients (32). These were mostly sedated and stable critically ill children. Additionally, our study population comprised a heterogeneous group of patients in terms of age, diagnosis, type, number, and severity of organ failures and type of ECMO. However, the study population was a representative sample of patients treated in our ECMO program and in many ECMO units around the world (with the exception of units that only use ECMO treatment in designated cardiac patients). Yet, it is possible our study did not have enough power to assess any change over time. Our sample size restricted our multivariable analyses, as we could not correct for all factors involved in these highly complex and critically ill patients. Nevertheless, we were able to explore which covariates possibly influenced the sublingual microcirculation. Future research is necessary to confirm these findings. Another limitation is that our observational study lacked a control group. However, it would have been unethical to randomly allocate severely ill patients to treatment with or without ECMO. Also, assessing whether the microcirculation was disturbed or not was difficult, since reference values for sublingual microcirculatory parameters are lacking. Finally, it should be noted that the sublingual microcirculation might not be reflective of microcirculatory beds of vital organs and this could be one of the explanations why sublingual microcirculatory parameters could not predict outcome. Yet the sublingual microcirculation was and remains an area of interest because of its easy accessibility for non-invasive measurements and alterations in this microcirculatory bed have been shown to be associated with increased morbidity and mortality rates in several different settings including ECMO patients (2, 33, 34).

To the best of our knowledge, this study is the first to look at the sublingual microcirculation throughout neonatal and pediatric ECMO treatment. Our study shows that the microcirculation is not solely dependent on global systemic hemodynamics and we have not yet elucidated this highly integrated network of vessels and its role in oxygen transport and tissue oxygenation. This study offers some insight in the applicability and feasibility of handheld vital microscopy in the neonatal and pediatric ECMO population. Our results suggest that the focus of future research should shift from routine monitoring of the microcirculation toward identifying patient populations that could benefit from microcirculatory monitoring, e.g., patients in cardiogenic shock. Evaluating the microcirculation in patients in cardiogenic shock could offer additional guidance for treatment strategies by showing whether inotropic and vasoactive drug support and/or mechanical support can sustain oxygen transport. More homogenous and larger study populations are needed to properly assess the effect of ECMO treatment on the microcirculation through multicenter studies focusing on certain subpopulations who receive ECMO treatment.

## Conclusion

In our heterogeneous study population, we were not able to demonstrate an effect of ECMO treatment on the sublingual microcirculation. The microcirculation was unaltered throughout ECMO treatment. Microcirculatory parameters did not differ between VV and VA ECMO or between survivors and non-survivors. Although monitoring of the microcirculation with handheld vital microscopy offers additional information on tissue oxygenation, its practice remains challenging in children. Future research should focus on determining which neonatal and pediatric ECMO patients would benefit from microcirculatory monitoring and how.

## Ethics Statement

We conducted an observational study on non-invasive microcirculatory monitoring. (Parental) informed consent was waived by our local medical ethical review board as standard therapy was monitored through non-invasive techniques in accordance with hospital guidelines.

## Author Contributions

ÖE, JK, JvR, DT, and CI contributed to the conception and design of the study. ÖE performed the inclusion of study patients, the microcirculatory measurements, the analysis of the microcirculatory imaging, and organized the database. ÖE, JK, and JvR performed the statistical analysis. ÖE and JK wrote the first draft of the manuscript. All authors contributed to manuscript revision, read, and approved the submitted version.

### Conflict of Interest Statement

CI has developed sidestream dark field imaging and is listed as an inventor on related patents commercialized by MicroVision Medical under a license from the Academic Medical Center in Amsterdam, the Netherlands. He has been a consultant for MicroVision Medical in the past, but has not been involved with this company for over 5 years now and holds no shares. Braedius Medical, a company owned by a relative of CI, has developed and designed a handheld microscope called CytoCam-IDF imaging used in this study. CI has no financial relationship with Braedius Medical of any sort, i.e., never owned shares, or received consultancy or speaker fees from Braedius Medical. The remaining authors declare that the research was conducted in the absence of any commercial or financial relationships that could be construed as a potential conflict of interest.

## Abbreviations

ECMO: extracorporeal membrane oxygenation
ECPR: extracorporeal cardiopulmonary resuscitation
HI: heterogeneity index
IS: inotrope score
FiO_2_: fraction inspired O_2_
MAP: mean arterial pressure
MFI: microcirculatory flow index
PaCO_2_: partial CO_2_ pressure
PaO_2_: partial O_2_ pressure
PELOD-2 score: pediatric logistic organ dysfunction 2 score
PPV: proportion perfused vessels
PVD: perfused vessel density
SMC: sublingual microcirculation
TVD: total vessel density
VA ECMO: venoarterial extracorporeal membrane oxygenation
VIS: vasoactive-inotrope score
VV ECMO: venovenous extracorporeal membrane oxygenation.

## References

[1] Ince C. Hemodynamic coherence and the rationale for monitoring the microcirculation. Crit Care. (2015) 19 (Suppl. 3):S8. 10.1186/cc14726 26729241 PMC4699073

[2] Top AP Ince C de Meij N van Dijk M Tibboel D. Persistent low microcirculatory vessel density in nonsurvivors of sepsis in pediatric intensive care. Crit Care Med. (2011) 39:8–13. 10.1097/CCM.0b013e3181fb7994 21076287

[3] Vellinga NA Boerma EC Koopmans M Donati A Dubin A Shapiro NI. International study on microcirculatory shock occurrence in acutely ill patients. Crit Care Med. (2015) 43:48–56. 10.1097/CCM.0000000000000553 25126880

[4] Scorcella C Damiani E Domizi R Pierantozzi S Tondi S Carsetti A. MicroDAIMON study: microcirculatory DAIly MONitoring in critically ill patients: a prospective observational study. Ann Intensive Care. (2018) 8:64. 10.1186/s13613-018-0411-9 29766322 PMC5953911

[5] Omar YG Massey M Andersen LW Giberson TA Berg K Cocchi MN. Sublingual microcirculation is impaired in post-cardiac arrest patients. Resuscitation. (2013) 84:1717–22. 10.1016/j.resuscitation.2013.07.012 23871865 PMC3864773

[6] Sakr Y Dubois MJ De Backer D Creteur J Vincent JL. Persistent microcirculatory alterations are associated with organ failure and death in patients with septic shock. Crit Care Med. (2004) 32:1825–31. 10.1097/01.CCM.0000138558.16257.3F 15343008

[7] Verdant CL De Backer D Bruhn A Clausi CM Su F Wang Z. Evaluation of sublingual and gut mucosal microcirculation in sepsis: a quantitative analysis. Crit Care Med. (2009) 37:2875–81. 10.1097/CCM.0b013e3181b029c1 19770750

[8] Kara A Akin S Dos Reis Miranda D Struijs A Caliskan K van Thiel RJ. Microcirculatory assessment of patients under VA-ECMO. Crit Care. (2016) 20:344. 10.1186/s13054-016-1519-7 27776535 PMC5078964

[9] Akin S Dos Reis Miranda D Caliskan K Soliman OI Guven G Struijs A. Functional evaluation of sublingual microcirculation indicates successful weaning from VA-ECMO in cardiogenic shock. Crit Care. (2017) 21:265. 10.1186/s13054-017-1855-2 29073930 PMC5658964

[10] Top AP Buijs EA Schouwenberg PH van Dijk M Tibboel D Ince C. The microcirculation is unchanged in neonates with severe respiratory failure after the initiation of ECMO treatment. Crit Care Res Pract. (2012) 2012:372956. 10.1155/2012/372956 22675619 PMC3366207

[11] Aykut G Veenstra G Scorcella C Ince C Boerma C. Cytocam-IDF (incident dark field illumination) imaging for bedside monitoring of the microcirculation. Intensive Care Med Exp. (2015) 3:40. 10.1186/s40635-015-0040-7 26215807 PMC4512989

[12] Ince C Boerma EC Cecconi M De Backer D Shapiro NI Duranteau J. Second consensus on the assessment of sublingual microcirculation in critically ill patients: results from a task force of the European Society of Intensive Care Medicine. Intensive Care Med. (2018) 44:281–99. 10.1007/s00134-018-5070-7 29411044

[13] De Backer D Hollenberg S Boerma C Goedhart P Buchele G Ospina-Tascon G. How to evaluate the microcirculation: report of a round table conference. Crit Care. (2007) 11:R101. 10.1186/cc6118 17845716 PMC2556744

[14] Massey MJ Larochelle E Najarro G Karmacharla A Arnold R Trzeciak S. The microcirculation image quality score: development and preliminary evaluation of a proposed approach to grading quality of image acquisition for bedside videomicroscopy. J Crit Care. (2013) 28:913–7. 10.1016/j.jcrc.2013.06.015 23972316

[15] Leteurtre S Duhamel A Salleron J Grandbastien B Lacroix J Leclerc F. PELOD-2: an update of the PEdiatric logistic organ dysfunction score. Crit Care Med. (2013) 41:1761–73. 10.1097/CCM.0b013e31828a2bbd 23685639

[16] Gaies MG Gurney JG Yen AH Napoli ML Gajarski R Ohye RG. Vasoactive–inotropic score as a predictor of morbidity and mortality in infants after cardiopulmonary bypass. Pediatr Crit Care Med. (2010) 11:234–8. 10.1097/PCC.0b013e3181b806fc 19794327

[17] Tuma RF Durán WN Ley K Handbook of physiology: Microcirculation. 2 ed. Boston, MA: Academic Press (2008). p. 1000.

[18] Top AP Ince C van Dijk M Tibboel D. Changes in buccal microcirculation following extracorporeal membrane oxygenation in term neonates with severe respiratory failure. Crit Care Med. (2009) 37:1121–4. 10.1097/CCM.0b013e3181962a5f 19237925

[19] Moscatelli A Pezzato S Lista G Petrucci L Buratti S Castagnola E. Venovenous ECMO for congenital diaphragmatic hernia: role of ductal patency and lung recruitment. Pediatrics. (2016) 138, E1–E5. 10.1542/peds.2016-1034 27940774

[20] McHoney M Hammond P. Role of ECMO in congenital diaphragmatic hernia. Arch Dis Child Fetal Neonatal Ed. (2018) 103:F178–F81. 10.1136/archdischild-2016-311707 29138242

[21] Kroth J Weidlich K Hiedl S Nussbaum C Christ F Genzel-boroviczeny O. Functional vessel density in the first month of life in preterm neonates. Pediatr Res. (2008) 64:567–71. 10.1203/PDR.0b013e318184134e 18596573

[22] Top AP van Dijk M van Velzen JE Ince C Tibboel D. Functional capillary density decreases after the first week of life in term neonates. Neonatology. (2011) 99:73–7. 10.1159/000316945 20733329

[23] van Elteren HA de Jonge RC van Rosmalen J Ince C Reiss IK. Adaptation of the cutaneous microcirculation in preterm neonates. Microcirculation. (2016) 23:468–74. 10.1111/micc.12295 27378187

[24] Wright IM Latter JL Dyson RM Levi CR Clifton VL. Videomicroscopy as a tool for investigation of the microcirculation in the newborn. Physiol Rep. (2016) 4:e12941. 10.14814/phy2.12941 27694527 PMC5064131

[25] Kuiper JW Tibboel D Ince C. The vulnerable microcirculation in the critically ill pediatric patient. Crit Care. (2016) 20:352. 10.1186/s13054-016-1496-x 27794361 PMC5086412

[26] Wu J Li C Yuan W. Phosphodiesterase-5 inhibition improves macrocirculation and microcirculation during cardiopulmonary resuscitation. Am J Emergen Med. (2016) 34:162–6. 10.1016/j.ajem.2015.09.033 26573781

[27] Buijs EA Reiss IK Kraemer U Andrinopoulou ER Zwiers AJ Ince C Increasing mean arterial blood pressure and heart rate with catecholaminergic drugs does not improve the microcirculation in children with congenital diaphragmatic hernia: a prospective cohort study. Pediatr Crit Care Med. (2014) 15:343–54. 10.1097/PCC.0000000000000105 24622167

[28] Zafrani L Ergin B Kapucu A Ince C. Blood transfusion improves renal oxygenation and renal function in sepsis-induced acute kidney injury in rats. Crit Care. (2016) 20:406. 10.1186/s13054-016-1581-1 27993148 PMC5168817

[29] Genzel-Boroviczeny O Christ F Glas V. Blood transfusion increases functional capillary density in the skin of anemic preterm infants. Pediatr Res. (2004) 56:751–5. 10.1203/01.PDR.0000141982.38959.10 15347769

[30] Schinagl CM Mormanova ZH Puchwein-Schwepcke A Schmid I Genzel-Boroviczeny O. The effect of red blood cell transfusion on the microcirculation of anemic children. Eur J Pediatr. (2016) 175:793–8. 10.1007/s00431-016-2704-z 26898704

[31] Yeh YC Lee CT Wang CH Tu YK Lai CH Wang YC. Investigation of microcirculation in patients with venoarterial extracorporeal membrane oxygenation life support. Crit Care. (2018) 22:200. 10.1186/s13054-018-2081-2 30121090 PMC6098836

[32] Gonzalez R Lopez J Urbano J Solana MJ Fernandez SN Santiago MJ. Evaluation of sublingual microcirculation in a paediatric intensive care unit: prospective observational study about its feasibility and utility. BMC Pediatr. (2017) 17:75. 10.1186/s12887-017-0837-5 28298202 PMC5353860

[33] Edul VS Enrico C Laviolle B Vazquez AR Ince C Dubin A. Quantitative assessment of the microcirculation in healthy volunteers and in patients with septic shock. Crit Care Med. (2012) 40:1443–8. 10.1097/CCM.0b013e31823dae59 22430243

[34] De Backer D Donadello K Sakr Y Ospina-Tascon G Salgado D Scolletta S. Microcirculatory alterations in patients with severe sepsis: impact of time of assessment and relationship with outcome. Crit Care Med. (2013) 41:791–9. 10.1097/CCM.0b013e3182742e8b 23318492

