# Supplementary material for: The Sublingual Microcirculation Throughout Neonatal and Pediatric Extracorporeal Membrane Oxygenation Treatment: Is It Altered by Systemic Extracorporeal Support?
Source: Front Pediatr. 2019 Jul 10;7:272. doi: 10.3389/fped.2019.00272 (PMC6636383; doi:10.3389/fped.2019.00272)
Supplement: Supplementary file 1 [file Table_1.docx]

**Supplementary material**

**Table S1.** Areas under ROC curves for mortality on ECMO and overall mortality

| **Outcome: Mortality on ECMO** | **Before start ECMO** | | | **After start ECMO/on day 1** | | | **ECMO day 2/3** | | |
| --- | --- | --- | --- | --- | --- | --- | --- | --- | --- |
|  | **AUC** | **95% CI** | **P-value** | **AUC** | **95% CI** | **P-value** | **AUC** | **95% CI** | **P-value** |
| TVD_all_ (mm/mm^2^) | 0.438 | 0.000-1.000 | 0.794 | 0.606 | 0.388-0.826 | 0.370 | 0.690 | 0.426-0.955 | 0.313 |
| PPV_all_ (%) | 0.536 | 0.000-1.000 | 0.794 | 0.583 | 0.355-0.812 | 0.480 | 0.405 | 0.101-0.708 | 0.614 |
| PVD_all_ (mm/mm^2^) | 0.500 | 0.000-1.000 | 1.000 | 0.617 | 0.396-0.838 | 0.322 | 0.714 | 0.450-0.978 | 0.257 |
| MFI_all_ | 0.688 | 0.000-1.000 | 0.794 | 0.589 | 0.356-0.822 | 0.451 | 0.250 | 0.000-0.509 | 0.186 |
| TVD_<20µm_ (mm/mm^2^) | 0.563 | 0.000-1.000 | 0.794 | 0.633 | 0.414-0.853 | 0.258 | 0.690 | 0.449-0.932 | 0.313 |
| PPV_<20µm_ (%) | 0.563 | 0.000-1.000 | 0.794 | 0.592 | 0.363-0.820 | 0.437 | 0.452 | 0.125-0.780 | 0.801 |
| PVD_<20µm_ (mm/mm^2^) | 0.563 | 0.208-1.000 | 0.433 | 0.661 | 0.453-0.869 | 0.172 | 0.690 | 0.452-0.929 | 0.313 |
| MFI_<20µm_ | 0.625 | 0.192-1.000 | 0.602 | 0.575 | 0.335-0.815 | 0.525 | 0.345 | 0.000-0.714 | 0.416 |
| **Outcome: Mortality** | **Before start ECMO** | | | **After start ECMO/on day 1** | | | **ECMO day 2/3** | | |
|  | **AUC** | **95% CI** | **P-value** | **AUC** | **95% CI** | **P-value** | **AUC** | **95% CI** | **P-value** |
| TVD_all_ (mm/mm^2^) | 0.286 | 0.000-0.756 | 0.305 | 0.544 | 0.332-0.757 | 0.690 | 0.692 | 0.446-0.939 | 0.258 |
| PPV_all_ (%) | 0.619 | 0.173-1.000 | 0.569 | 0.706 | 0.507-0.907 | 0,063 | 0.500 | 0.201-0.799 | 1.000 |
| PVD_all_ (mm/mm^2^) | 0.333 | 0.000-0.867 | 0.425 | 0.574 | 0.364-0.783 | 0,507 | 0.712 | 0.470-0.953 | 0.213 |
| MFI_all_ | 0.619 | 0.216-1.000 | 0.569 | 0.623 | 0.412-0.833 | 0,268 | 0.269 | 0.030-0.509 | 0.174 |
| TVD_<20µm_ (mm/mm^2^) | 0.333 | 0.000-0.867 | 0.425 | 0.539 | 0.322-0.756 | 0,723 | 0.769 | 0.547-0.991 | 0.113 |
| PPV_<20µm_ (%) | 0.619 | 0.173-1.000 | 0.569 | 0.713 | 0.515-0.911 | 0,054 | 0.558 | 0.244-0.871 | 0.734 |
| PVD_<20µm_ (mm/mm^2^) | 0.333 | 0.000-0.867 | 0.425 | 0.583 | 0.373-0.794 | 0,452 | 0.750 | 0.519-0.981 | 0.141 |
| MFI_<20µm_ | 0.571 | 0.191-0.951 | 0.732 | 0.586 | 0.372-0.779 | 0,438 | 0.356 | 0.055-0.657 | 0.396 |

*AUC = area under curve; CI = confidence interval; ECMO = extracorporeal membrane oxygenation; MFI = microcirculatory flow index; PPV = proportion perfused vessels; PVD = perfused vessel density; ROC = receiver operational characteristic; SD = standard deviation; TVD = total vessel density.*
